# Hepatocyte and Islet Cell Cotransplantation on Poly-L-Lactide Matrix for the Treatment of Liver Cirrhosis

**DOI:** 10.1155/2020/5410359

**Published:** 2020-10-13

**Authors:** Siufui Hendrawan, Jennifer Lheman, Nuraeni, Ursula Weber, Hans Ulrich Baer

**Affiliations:** ^1^Tarumanagara Human Cell Technology Laboratory, Faculty of Medicine, Tarumanagara University, Jakarta 11440, Indonesia; ^2^Department of Biochemistry and Molecular Biology, Faculty of Medicine, Tarumanagara University, Jakarta 11440, Indonesia; ^3^Baermed, Centre of Abdominal Surgery, Hirslanden Clinic, 8032 Zürich, Switzerland; ^4^Department of Visceral and Transplantation Surgery, University of Bern, Switzerland

## Abstract

The human autologous hepatocyte matrix implant is a promising alternative procedure to counter liver damage. We assessed the outcome of human hepatocytes isolation from cirrhotic liver compared to the clinical and histological scores of disease severity. A total of 11 patients with various clinical scores (CTP and MELD) and histological score (Metavir, fibrosis) of liver cirrhosis were included in the hepatocyte matrix implant clinical phase I study. The liver segment and pancreatic tissue were harvested from each patient, and hepatocytes and cells of islets of Langerhans were isolated. The freshly isolated human hepatocytes were coseeded with the islet cells onto poly(l-lactic acid) (PLLA) scaffolds, cultured, and transplanted back into the patient. Human hepatocytes were isolated from 11 cirrhotic liver specimens with a resulting yield of 1.4 ± 0.5 × 10^6^ cells per gram of the liver specimen and a viability rate of 52 ± 13%. It was found that the yield and viability of the liver cells were not correlated with the clinical and histological scores of the liver cirrhosis. A correlation was found between the hepatocyte yield obtained and the average number of hepatocytes counted in 10 microscopic fields of view. More viable cells were obtained from cirrhotic livers caused by chronic hepatitis B as compared to chronic hepatitis C in the same MELD score range. There was no correlation between the clinical and histological disease severity scores of liver cirrhosis and the outcome of hepatocytes isolation. It seems that the yield could depend on the type of hepatitis underlying the cirrhotic tissue. The study was registered at www.clinicaltrial.gov with the study identifier: NCT01335568.

## 1. Introduction

Many patients die due to liver failure from diverse causes. Even though conventional drug therapy may help to delay the progression or reduce symptoms of the disease, it is often unable to restore the function of the damaged tissue completely. For that reason, transplantation of a healthy liver from a suitable donor is for many patients the only definitive treatment option. However, the lack of suitable organ donors remains a major obstacle. While the supply of cadaver donor organs has been constant for a decade, demand for transplantable livers has been progressively increasing, outpacing the availability of the donor organs [1]. Furthermore, organ transplant programs may not be available in many developing countries.

In an attempt to find alternative treatment methods for cirrhotic disease, a novel cell-based approach as hepatocyte transplantation was found [2]. This procedure has gained importance as an alternative or supportive treatment to orthotropic liver transplantation (OLT). It could offer a possible solution to the organ shortage. We have established the intracorporeal autologous hepatocyte matrix implant (HMI) as a possible therapeutic approach for patients with liver cirrhosis that offers these patients a form of definite treatment or bridging therapy (Bridge-To-Transplant) [3]. The implant may help to stabilize and improve the liver function until an organ for transplant becomes available. It may also serve as a definitive treatment; the HMI procedure offers one big advantage as it uses the patient's own liver tissue as the cell source. Immunosuppressive therapy is not necessary because of the autologous nature of the hepatocytes.

In this procedure, pancreatic cells were coseeded to stimulate hepatocyte growth. It has been found that the proliferation and survival of hepatocytes can be stimulated by cocultivation with cells of islets of Langerhans. Particularly, the beta cells (insulin-producing cells) were found to increase the growth rate of hepatocytes [4]. Therefore, these transplanted hepatocytes, by adding cotransplantation of islet cells, may contribute to improve the failing liver's function. However, this cellular approach may depend on cell sources, cell mass, and viability, considering the difficulties in isolation and cultivation of primary hepatocytes from damaged tissue. This study assessed factors that predict hepatocyte isolation outcomes (yield and viability) in liver cirrhosis patients. We compared the clinical disease severity and the grading of liver cirrhosis of the patient before entering the study with the yield of human hepatocyte obtained from the liver segment.

## 2. Methods

### 2.1. Autologous Hepatocyte Matrix Implant (HMI) Procedure

All procedures in the phase I study were performed according to the protocol approved by the local Research Ethics Committee, ethical approval number #REP2010_001. Surgical procedures were carried out at the Gading Pluit Hospital, Jakarta, Indonesia. Liver tissues were obtained from fully consenting patients undergoing the HMI procedure with a variety of end-stage liver disease. In brief, the procedure includes harvesting the patient's own liver and pancreatic tissue in the first operation. The hepatocytes and islets of Langerhans cells were then isolated from these tissues, by mechanical and enzymatic methods. The cells were cocultured and seeded on biodegradable PLLA scaffolds. In a second operation, these engineered cell-scaffolds were implanted back into the patient's small bowel mesentery [3].

### 2.2. PLLA Scaffold Preparation

The matrix is a three-dimensional (3D) porous scaffold that comprises poly (l-lactic acid) (PLLA) polymers. The scaffolds were obtained from Phrontier SARL, France, and treated with a plasma sterilization system (Johnson & Johnson, USA). It is a 3D circular-shaped sponge with a diameter of 2 cm and a thickness of 4 mm with the porosity around 90%. Prior to its use, the sterilized scaffold was precoated with 0.1% collagen solution type-I from calf skin (Sigma, USA) to facilitate cell seeding and attachment [5].

### 2.3. Hepatocyte Isolation

Human hepatocytes were isolated from 11 harvested cirrhotic liver specimens. A 4 cm × 4 cm × 4 cm liver segmental tissue and a 3 mm × 3 mm × 3 mm tissue from the pancreas were harvested and immediately placed in a Custodiol® transplant solution and triple-bag sterile packaging with crushed ice, then transported to the Tarumanagara Human Cell Technology (THCT) Laboratory in Jakarta, to be processed within 2-3 hours.

Hepatocyte isolation was carried out using a modified two-stage collagenase perfusion technique [6, 7]. After the sterility test, the liver segment was weighed and cut to identify suitable vessels for cannulation. The perfusion circuit was set up, with one end of the perfusion tube fed into the perfusion solution, the central segment of the tubing was within the peristaltic pump (Ismatec MCP process IP65, Cole-Parmer, Germany), and the other end of the tubing was connected to the cannulae. Subsequently, six 22-gauge cannulae (B. Braun, Germany) were inserted into the vessels. During the perfusion, the perfusion solutions were purged by washed carbon gas via a sterile filter (0.22 *μ*m pore size). All solutions used in the perfusion were prewarmed to 37°C in a water bath. The liver segment was first perfused with 1 L wash buffer Ethylene Glycol Tetra-acetic Acid (EGTA) (Sigma, USA) using flow rates of 20-60 rpm, to flush out any remaining blood. After this, the segment was perfused with 900 mL enzyme solution (5 U/mL) containing collagenase (Sigma, USA) and hyaluronidase (Sigma, USA) to dissociate the liver using flow rates 60 rpm until the liver piece softened and turned lighter in color. The liver segment was then cut into fine pieces using a tissue chopper and soaked in an enzyme solution (10 U/mL) containing collagenase and hyaluronidase. Following the mechanical dissociation of the liver segment, the suspensions were passed through a tea strainer (0.8 mm mesh size), followed by a cell sieve of 100 *μ*m mesh size (Retsch, Germany) with the aid of a sterile cell scraper. Suspensions were then washed three times at 130 G for 10 minutes in William E medium (Sigma, USA) supplemented with fetal bovine serum (FBS) (Sigma, USA). After the final wash, the cell pellet was suspended in William E medium supplemented with 10% v/v of the patient's autologous serum. The cell number and viability were determined by trypan blue (Sigma, USA) dye exclusion method using a haemacytometer.

### 2.4. Pancreatic Single Cell Isolation

Islet of Langerhans cells were isolated in parallel with hepatocytes isolation as described in the literature [7]. The pancreatic tissue was weighed and cut with sterile scalpels in a petri dish filled with 10 mL enzyme solution (10 U/mL). The dissociated pieces were then incubated at 37°C with 5% CO_2_ for 15 minutes. Following the incubation period, the suspension was passed through a tea strainer. Suspensions were then washed once with 10-15 mL William E medium (Sigma, USA) supplemented with FBS (Sigma, USA) at 130 G for 10 minutes, and the last cell pellet was resuspended in William E medium supplemented with 10% v/v of autologous serum. The assessment of yield and viability was also performed using a trypan blue exclusion assay. The pancreatic cell suspension was then mixed with hepatocyte suspension.

### 2.5. Cell Seeding and Culture on Scaffolds

The PLLA scaffolds were placed in a 12-well plate and seeded with a 300 *μ*L cell suspension (the mixture of isolated hepatocytes and islet cells) with an average density of 1.5 × 10^6^ cells per scaffold, and 0.6-1 mL warm medium was added in each well. Engineered cell-scaffolds were cultivated at 37°C with 5% CO_2_ overnight. On the next day, the culture medium was replaced with a fresh medium; cells not attached to the matrices were collected from media and counted. Around 63 hours after the seeding, the scaffolds and cells adherent thereto were transferred into a new 12-well plate and filled with 0.3 mL prewarmed William E medium supplemented with 10% v/v autologous serum. The remaining cells in the previous wells were collected and counted. The engineered cell-scaffolds were transported back to the hospital in a monitored thermal box (35-37°C) for implantation into the patient.

### 2.6. Histological Morphological Assessment

A tissue sample additionally harvested during the liver tissue harvesting was preserved for histological assessment. The sections of the specimens were stained with hematoxylin and eosin. The Metavir scoring system was used to assess the extent of inflammation and fibrosis by histopathological evaluation in a liver segment of the patients. The number of hepatocytes was counted in 10 random microscopic fields of view.

### 2.7. Data Analysis and Statistical Methods

The results are expressed as means ± SD. The normal distribution of the data was assessed using the Shapiro-Wilk test. The correlation between variables was determined by two-tailed Pearson's test, and the values were considered statistically significant at *p* ≤ 0.05.

## 3. Results

50 liver cirrhosis patients were evaluated, and according to the inclusion criteria of the trial, 11 patients were included, 3 patients with chronic hepatitis B, 7 patients with chronic hepatitis C, and 1 patient with nonviral chronic hepatitis. At the initiation of the procedure, patients displayed a Child-Turcotte-Pugh (CTP) score between 6 and 9 and a MELD score between 9 and 21. Histological assessments of each liver cirrhotic specimen showed a Metavir activity grade of between 2 and 3 and fibrosis of stage 4 for all the patients. The inclusion criteria excluded patients with active viral hepatitis. We included patients with viral load up to 3 × 10^5^ IU/mL, but also three hepatitis patients with undetectable viral counts. In the case of hepatitis B, we were able to rely on oral antivirus medication. The clinical outcomes, laboratory parameters, and the impact of viral load have been reported in our first publication. Some patients showed improvement in clinical condition, reduced frequency of hospitalization, and reduced incidences of liver cirrhosis complications [3]. The average number of hepatocytes in 10 fields of view was 40 ± 15 cells (Table 1).

Hepatocytes were processed and isolated from 11 cirrhotic liver specimens with a total cell yield of 10 × 10^6^ to 64 × 10^6^ cells per liver specimen and a mean viability rate of 52 ± 13% (Table 2).

The yield of isolated hepatocytes per gram specimen was compared to clinical and histological disease severity scores. The mean hepatocyte yield was 1.4 ± 0.5 × 10^6^ per gram tissue (Table 2).

The correlation between hepatocyte yield per gram liver tissue, clinical and histological scores, and the average number of hepatocytes in 10 fields of view was analyzed statistically. The yield and viability of the liver cells were not found to be correlated with the clinical and histological scores of the liver cirrhosis. There was no significant correlation between the hepatocyte yield obtained and the patient's CTP score, MELD score, Metavir activity grade, or fibrosis stage of the cirrhotic tissue. There was also no correlation found between the cell viability and these scores. The results showed that the only significant correlation (*p* < 0.05) was between the cell yield obtained and the average number of normal hepatocytes in 10 fields of view. More viable cells were obtained from cirrhotic livers caused by chronic hepatitis B as compared to chronic hepatitis C in the same MELD score range.

The harvested pancreatic tissue samples were also treated to liberate the pancreatic cells. The average amount of pancreatic cells was 82,091 ± 108,876 cells with a mean viability of 89 ± 9%. Cells, after quantification, were coseeded onto the matrices. For each patient, approximately 17 engineered cell-scaffolds were generated and implanted. Not all the cells that were seeded on the matrices were attached to the matrices. Dead cells and some viable cells that could not adhere to the matrices were found in the leftover suspension at the multiwell plate. The mean of cell adhesion rate on the matrices was 72 ± 11%.

## 4. Discussion

Hepatocyte transplantation is a potential alternative or bridge to liver transplantation. Numerous clinical trials of hepatocyte transplantation in the last two decades indicate that transplants consisting of isolated liver cells can correct liver diseases such as cirrhosis, acute liver failure, and metabolic liver disease and provide restorative potential in case of liver failure [8]. Although clinical results are encouraging, hepatocyte transplantation still faces limitations, mainly concerning the limited source of donor hepatocytes and the lack of a long-lasting effect. These issues need to be overcome to broaden its clinical applications. To date, hepatocyte functions can be improved through coculturing with mesenchymal stem cells (MSC) [9]. Furthermore, Yimlamai et al. [10, 11] discovered new evidence in a mouse model that “the Hippo-signaling pathway is an important regulator of cellular proliferation and organ size.” He showed that through this signaling pathway, mature hepatocytes can be induced to revert to a stem cell-like state. Yimlamai reports that “Remarkably, acute inactivation of Hippo pathway signaling in vivo is sufficient to dedifferentiate, at very high efficiencies, adult hepatocytes into cells bearing progenitor cells” [10, 11].

Hepatocyte transplantation for end-stage liver disease is even more problematic. Transplantation of hepatocytes into the portal vein of a cirrhotic liver can generate severe portal hypertension [12]. The intraportal infusion generally used a high number of hepatocytes. However, although it used a high number of cells, the exact number of cells engrafted and function immediately to reverse hepatic failure remains essentially unknown [13]. The extrahepatic site has been suggested as an alternative to intraportal injection in patients with liver cirrhosis, in whom altered preexisting fibrosis liver architecture could impact hepatocyte engraftment [14]. Alpha-1 antitrypsin was proven to improve the engraftment of the transplanted hepatocytes; however, it has not been studied at a clinical level [15].

Our HMI procedure represents a promising approach for coculturing human hepatocytes with cells of islets of Langerhans and preparing them for implantation on a biodegradable polymer matrix into the mesentery leaves. Vascularization of the implants is crucial for a meaningful tissue function. The mesentery of the small bowel has been found to provide a suitable implant location to establish vascularization and integration [16]. We relied on the data of Kneser who has implanted hepatocytes cotransplanted with cells of islets on scaffolds between the mesenteries of the rat's small bowel [17]. We speculate that splanchnic blood draining from the pancreas and other intestinal organs could transport important growth factors to the cells on the scaffolds at this place. Through this approach, a sufficient hepatocyte cell mass could be implanted into patients' own bodies to support liver function, and since it will be autologous in nature, patients will not face immune rejection. This could overcome the technical problem related to intraportal infusion, a restriction on the number of transplantable cells, and insufficient cell survival and engraftment [18, 19]. Our HMI clinical phase I study was primarily designed to prove the safety of the procedure. The clinical outcomes have been reported in our first publication; most patients did experience a general improvement in condition [3].

The outcome of hepatocyte transplantation will depend on the quality and number of cells transplanted. Primary hepatocytes have been utilized as the cell source for hepatocyte transplantation. Many published studies have reported the successful outcomes of human hepatocyte isolation from normal liver tissue [18]. However, cirrhotic livers could also be a valuable source of human hepatocytes. In our study, we isolated hepatocytes from fibrotic livers, from cirrhosis patients, caused by chronic hepatitis. The isolation of human hepatocytes from diseased liver tissue has been difficult with overall results so variable in terms of yield and viability of cells [20]. We established the human hepatocytes isolation method from cirrhotic liver tissue with a total cell count of 10 × 10^6^ to 64 × 10^6^ cells per liver specimen and a mean viability rate of 52 ± 13%. Given the autologous nature of the HMI procedure, the number of isolated cells per patient varied significantly. The physical condition of the harvested liver tissue was also different. Bhogal et al. reported that liver tissue from patients with biliary cirrhosis provides median hepatocyte viability of 55%; this is in line with our outcome [6]. They also reported that the most important factor affecting successful human hepatocyte isolation was the time delay between liver resection and the beginning of liver perfusion. In our case, the tissues were freshly processed within 2-3 hours after the resection, resulting in higher cell yield compared to the result that has been reported by Bhogal et al. [6].

We found no correlation between the clinical disease severity (CTP and MELD) of the patients before entering the study or the grading of liver cirrhosis (Metavir) with the yield of human hepatocytes obtained from the liver segment. Another study showed that liver cirrhosis does not have any effect on yield and the viability of the hepatocytes; however, the result could not be concluded because they only had 4 patients with liver cirrhosis [21]. As reported by Richert et al., the success of human hepatocyte isolation is not significantly affected by preoperative factors such as age, sex of the patient, steatosis, or cholestasis. Protocols in terms of tissue collection and transport, liver biopsy weight, cannulation, and isolation procedure should optimize the total yield of viable human hepatocytes obtained per preparation of the liver segment [22]. No studies are available, comparing the outcome of human hepatocyte isolation and the number of hepatocytes in microscopic fields. In our study, we showed that there is a correlation between the number of hepatocytes counted in the microscopic field of view and the yield of hepatocytes isolated from the tissue. A higher number of normal hepatocytes as counted in a microscope field of view was found to be associated with a higher yield of hepatocytes obtained.

We expect that the intracorporeal autologous hepatocyte matrix implant could meet the metabolic requirements of the recipient. The new tissue must be alive as well as its functionality must meet both the qualitative and quantitative requirements of the respective transplant indication. The long-term survival characteristics of the bioartificial liver tissue have to be guaranteed. Previous animal studies have shown that hepatocytes when seeded into 3D PLA scaffolds, implanted into rats, were fully viable and functional for at least six to twelve months [17, 23]. The first results in Germany showed that a significant improvement in liver function and general health condition was proven in nine out of ten patients [7]. In Germany, liver cirrhosis commonly caused by alcoholism [24] differs from liver cirrhosis in Indonesia that is more often caused by the hepatitis virus [25]. Hepatitis B or C virus causes the damage of tissue scattered throughout the organ. It may explain the reason for the yield and viability of liver cells obtained in our study to be lower than that found in the study in Germany. There was no direct correlation between the stage of cirrhosis to the yield and viability of the liver cells. On the other hand, we obtained more viable cells from chronic hepatitis B patients as compared to chronic hepatitis C patients with the same level of MELD score. HMI may serve as a valuable alternative procedure of hepatocyte transplantation for liver cirrhosis patients. We are now conducting a phase II study with more patients to prove the efficacy of this study.

## 5. Conclusions

There was no direct correlation between the stage of liver cirrhosis to the yield and viability of the liver cells. The cause of cirrhosis (the type of hepatitis) can alter the yield and viability of isolated liver cells.

## Figures and Tables

**Table 1:** Patient baseline assessment including histological results of the 11 patients.

	Gender	Age at study entry	Diagnosis	Viral load (IU/mL)	Clinical scores		Histological score	Average number of hepatocytes in 10 fields of view
					CTP score	MELD score	Metavir^∗^	
Median (range)	64% M	58 (43-67)		6.4 × 10^3^ (9.5 to 3 × 10^5^)	8 (6-9)	15 (9-21)	A3, F4 (A2-A3; F4)	40 (19-61)
	Patients				Hep (cirrhosis)			
1	M	58	Hep. C	Undetected	8	15	A3, F4	61
2	M	52	Hep. B	9.56 × 10^0^	8	16	A2, F4	26
3	M	56	Hep. B	8.44 × 10^2^	8	12	A3, F4	52
4	M	57	Hep. C	Undetected	8	12	A2, F4	51
5	F	67	Hep. C	3.56 × 10^3^	6	10	A2, F4	59
6	M	43	Hep. C	9.22 × 10^3^	6	11	A2, F4	49
7	F	58	Nonviral	N.A	9	21	A3, F4	19
8	F	59	Hep. C	3.01 × 10^5^	6	9	A3, F4	33
9	F	60	Hep. C	1.47 × 10^5^	9	15	A3, F4	20
10	M	59	Hep. C	1.6 × 10^5^	9	18	A3, F4	29
11	M	56	Hep. B	1.05 × 10^5^	8	16	A2, F4	40

^∗^Metavir score: A: activity, inflammation; F: fibrosis.

**Table 2:** Isolation of human hepatocytes from liver segments of the patients.

Patients	Hepatocytes isolation			
	Tissue weight (g)	Total cell yield	Cell yield per gram	Viability
1	11.8	24,000,000	2.0 × 10^6^	68%
2	32.4	38,000,000	1.2 × 10^6^	56%
3	31.3	63,800,000	2.0 × 10^6^	62%
4	15.8	30,000,000	1.9 × 10^6^	62%
5	17.5	34,000,000	1.9 × 10^6^	66%
6	18.1	13,000,000	0.7 × 10^6^	34%
7	10.5	9,600,000	0.9 × 10^6^	40%
8	17.4	20,000,000	1.1 × 10^6^	40%
9	14.9	20,000,000	1.3 × 10^6^	33%
10	19.6	15,000,000	0.8 × 10^6^	50%
11	17.4	28,000,000	1.6 × 10^6^	60%

## Data Availability

The data used to support the findings of this study are included within the article.
